# Multiparametric cardiovascular magnetic resonance characterization of a rare chordoma metastasis to the heart

**DOI:** 10.1093/ehjci/jeaf040

**Published:** 2025-02-13

**Authors:** Stefano Figliozzi, Kamil Stankowski, Lorenzo Monti, Marco Francone

**Affiliations:** Department of Cardiovascular Diseases, IRCCS Humanitas Research Hospital, Via Alessandro Manzoni, 56, Rozzano 20089, Milano, Italy; Department of Biomedical Sciences, Humanitas University, Via Rita Levi Montalcini, 4, Pieve Emanuele 20090, Milano, Italy; Department of Cardiovascular Diseases, IRCCS Humanitas Research Hospital, Via Alessandro Manzoni, 56, Rozzano 20089, Milano, Italy; Department of Biomedical Sciences, Humanitas University, Via Rita Levi Montalcini, 4, Pieve Emanuele 20090, Milano, Italy; Department of Diagnostic and Interventional Radiology, IRCCS Humanitas Research Hospital, Via Alessandro Manzoni, 56, Rozzano 20089, Milano, Italy; Department of Biomedical Sciences, Humanitas University, Via Rita Levi Montalcini, 4, Pieve Emanuele 20090, Milano, Italy; Department of Diagnostic and Interventional Radiology, IRCCS Humanitas Research Hospital, Via Alessandro Manzoni, 56, Rozzano 20089, Milano, Italy

**Figure:**
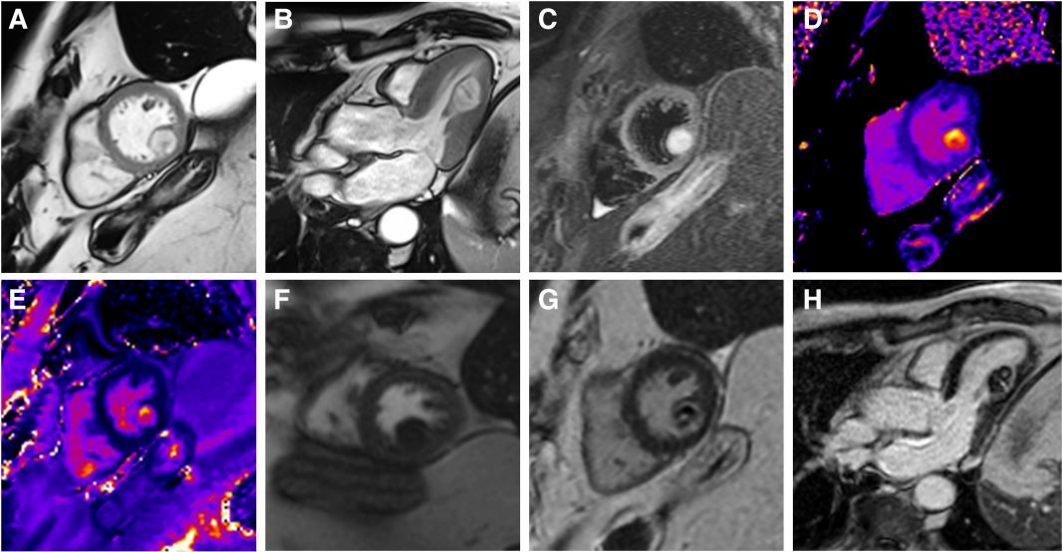


In 2010, a 68-year-old man underwent coccyx resection and radiotherapy because of localized sacral chordoma. In 2015, he received a left superior pulmonary lobectomy because of chordoma metastasis. In 2019, cardiovascular magnetic resonance (CMR) detected a mass at the base of the posteromedial papillary muscle (15 × 14 mm;Supplementary data online, *Figure S1*). In 2023, the patient, still asymptomatic, was referred to us for follow-up CMR because of concerns of mass growth at the latest echocardiography (see Supplementary data online, *Figure S2*, Supplementary data online, *Video S1*). An oval mass contained within the posteromedial papillary muscle with well-defined margins and increased dimensions (26 × 18 mm), as compared with the previous CMR, was seen. In comparison to the myocardium, the mass was hyperintense in cine-SSFP (*Panels A* and *B*; Supplementary data online, *Videos S2* and *S3*), markedly hyperintense in the T2-weighted STIR (*Panel C*) and showed increased T1 and T2 mapping values (*Panels D* and *E*). After contrast injection, there was no increased mass signal at first-pass perfusion (*Panel F*), whereas the late gadolinium sequences revealed soft, heterogeneous enhancement (*Panels G* and *H*). The CMR properties of the mass were unchanged compared with the previous scan and were interpreted as a growing cardiac metastasis of sacral chordoma. Treatment with imatinib was initiated.

Chordomas are rare tumours of the axial skeleton. Chordomas tend to metastasize, but cardiac metastases are rare and demonstrate very slow growth. CMR is well-suited for the characterization of cardiac masses. The present case represents the first description of a chordoma metastasis within a papillary muscle and the first to provide multiparametric CMR characterization of a cardiac metastasis secondary to this tumour.

Supplementary data are available at *European Heart Journal - Cardiovascular Imaging* online.

**Funding:** This work was partially supported by “Ricerca Corrente” funding from Italian Ministry of Health to IRCCS Humanitas Research Hospital.

**Data availability:** No new data were generated or analysed in support of this research.

## Supplementary Material

jeaf040_Supplementary_Data

