# Supplementary figures and images for: Multiparametric cardiovascular magnetic resonance characterization of a rare chordoma metastasis to the heart
Source: Eur Heart J Cardiovasc Imaging. 2025 Feb 13;26(6):1075. doi: 10.1093/ehjci/jeaf040 (PMC12123508; doi:10.1093/ehjci/jeaf040)

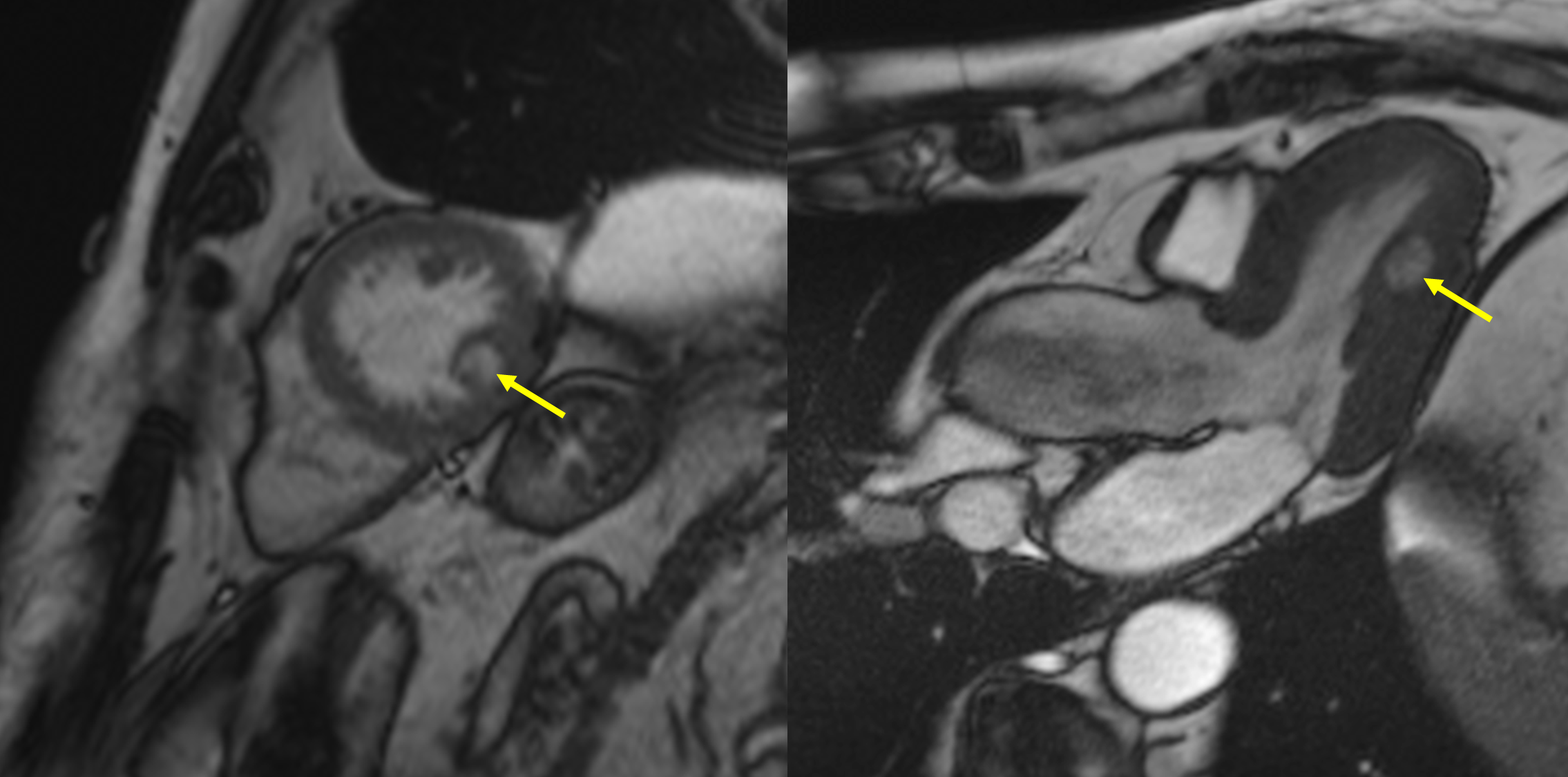

Supplement: jeaf040_Supplementary_Data [file jeaf040_supplementary_data.zip › Supplementary Figure 1.tif]

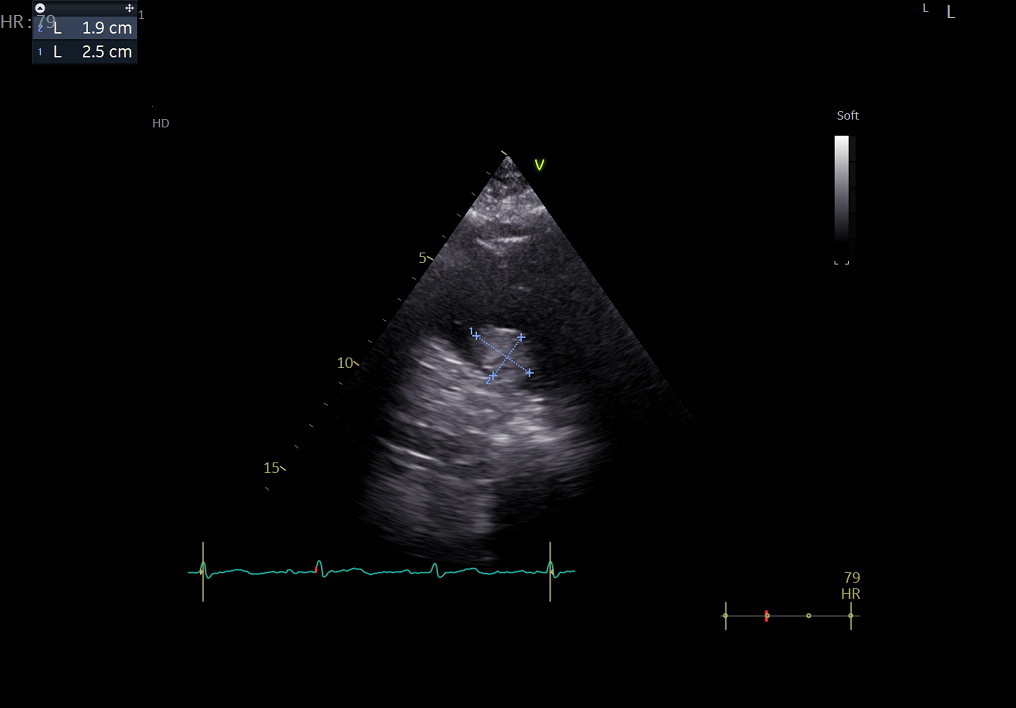

Supplement: jeaf040_Supplementary_Data [file jeaf040_supplementary_data.zip › Supplementary Figure 2.jpg]
